# SMAD4 Protein Alterations in Early-Onset Colorectal Cancer: Implications as a Potential Marker for Aggressive Disease and Prognosis—A Clinicopathological and Molecular Analysis of 18 Cases in Patients < 40 Years of Age

**DOI:** 10.3390/diagnostics16121804

**Published:** 2026-06-11

**Authors:** Lingling Xian, Jim Lu, Lan Zhou, Wei Xin

**Affiliations:** 1 Department of Pathology, University of South Alabama, Mobile, AL 36617, USA; lingling.xian@yale.edu; 2 Department of Pathology, Yale University School of Medicine, New Haven, CT 06520, USA; 3 GoPath Diagnostics, Buffalo Grove, IL 60089, USA; jlu@gopathdx.com; 4 Weill Cornell Medical College, Cornell University, Huston, TX 14850, USA; lzhou3@houstonmethodist.org

**Keywords:** early-onset colorectal cancer, SMAD4, genetic alterations, protein expression, metastasis, survival

## Abstract

**Background/Objectives:** Colorectal cancer (CRC) is relatively uncommon in individuals under 40 years of age; however, its rising incidence is a growing concern. This study aimed to investigate clinicopathologic features, genetic alterations, and protein expression patterns in early-onset colorectal cancer (EOCRC) to better understand the underlying mechanisms and prognostic factors. **Methods:** We retrospectively analyzed 18 patients diagnosed with EOCRC (<40 years) at our institution between 2018 and 2023. Next-generation sequencing (NGS) and immunohistochemistry (IHC) were used to assess genomic alterations and protein expression profiles. Clinicopathologic data were correlated with molecular findings and outcomes. **Results:** The cohort included ten females and eight males (mean age, 32.7 years; range, 17–38 years). Tumors most frequently arose in the rectum (56%) and were predominantly high stage (T3–T4, 67%) and moderately differentiated (78%). Lymphovascular invasion occurred in 50% of cases, and lymph node metastasis in 39%. Most tumors were microsatellite stable (MSS, 89%) and mismatch repair–proficient; two cases (11%) were MSI-high with germline MMR mutations. Among 17 patients who underwent NGS, the most frequent mutations involved KRAS (35%), APC (24%), TP53 (18%), and SMAD4 (18%). Notably, SMAD4 protein downregulation by IHC was observed in 67% of cases, including 60% of SMAD4 wild-type tumors. Loss of SMAD4 expression was significantly associated with lymph node metastasis (*p* = 0.037) and poor survival (*p* = 0.045). **Conclusions:** SMAD4 alteration—on both the genetic and protein levels—is common in EOCRC and is significantly correlated with aggressive clinicopathologic features and worse prognosis.

## 1. Introduction

Colorectal cancer (CRC) is the third most diagnosed malignancy worldwide and the second leading cause of cancer-related mortality [1], affecting individuals over the age of 50. The incidence of CRC in individuals under the age of 50, known as early-onset colorectal cancer (EOCRC), has been on the rise [2], drawing attention to its unique clinicopathologic and molecular characteristics. Unlike older patients, EOCRC patients, especially those under 40 years, are less likely to undergo routine colonoscopy screening, leading to delays in diagnosis and potentially more aggressive disease at presentation [3,4]. Lifestyle factors, environmental exposures, and genetic predispositions are thought to contribute to this trend [5].

While mutational and transcriptional profiles in EOCRC have been previously investigated [6,7,8], limited attention has been given to changes at the protein level. Of particular interest is the SMAD4 gene, a crucial component of the TGF-β signaling pathway, which has garnered attention due to its established role in juvenile polyposis syndrome (JPS), CRC, and other hereditary tumors in young patients [9]. Juvenile polyposis syndromes with mutations in the SMAD4 and BMPR1A genes confer a 10–38% lifetime risk of CRC [10,11]. Alterations in SMAD4, such as genomic mutations and loss of protein expression, have been associated with advanced disease, metastasis, and poor prognosis in late-onset CRC patients [12,13]. SMAD4 mutations were detected in 14.3 to 19% EOCRC in some studies [6,14]; however, the status of SMAD4 protein expression in EOCRC remains to be elucidated.

In this study, we retrospectively examined clinicopathologic features, genetic alterations, and protein expression in 18 patients diagnosed with EOCRC under the age of 40 at our institution from 2018 to 2023. Although EOCRC is generally defined as colorectal cancer diagnosed before the age of 50 years, we selected a cutoff of <40 years to enrich for a very young EOCRC population, in whom hereditary predisposition and unique disease characteristics are more prevalent than in the broader EOCRC population defined by age < 50 years. Utilizing next-generation sequencing (NGS) and immunohistochemistry (IHC), we particularly focused on SMAD4 genomic mutations and the patterns of protein expression, aiming to uncover their clinical significance and association with disease prognosis in this unique population. Our findings reveal a profound alteration in SMAD4 protein level, which may serve as a potential marker for aggressive disease phenotypes and poor clinical outcomes in young CRC patients, thereby providing insights into the biological behavior of EOCRC and suggesting novel avenues for therapeutic intervention.

## 2. Materials and Methods

### 2.1. Case Selection and Data Collection

This study was approved by the Institutional Review Board of the University of South Alabama, Whiddon College of Medicine. A HIPAA waiver was granted, as the research involved a discarded tissue and chart review study with no collection or storage of patient-identifiable data post-screening. A computer search of the surgical pathology files of the University of South Alabama, Whiddon College of Medicine, was carried out (encompassing the years 2018 to 2023) for colon adenocarcinoma in individuals under 40 years old; eighteen cases were identified, and they were all surgical resections.

All specimens were formalin-fixed and paraffin-embedded tissue sections. Hematoxylin and eosin-stained slides were reviewed for confirmation. Detailed clinicopathological data, including tumor diagnosis, TNM classification, tumor location, mismatch repair (MMR) status, Microsatellite Instability (MSI) test, NGS, and patient follow-up status, were extracted from the pathology and clinical records. Survival times were obtained and correlated with medical record charts for data analysis (Table 1). All patients underwent surgical resection as primary treatment. Adjuvant chemotherapy was administered based on tumor stage and institutional clinical guidelines; however, detailed treatment regimens were not uniformly available due to the retrospective nature of the study.

### 2.2. Next-Generation Sequencing (NGS)

Formalin-fixed paraffin-embedded (FFPE) samples underwent next-generation sequencing (NGS) to assess genetic alterations. To ensure quality, an independent pathologist reviewed hematoxylin and eosin (H&E) stained sections to confirm a >50% tumor content in each sample. All sample preparation, NGS, and bioinformatics analyses were conducted in a CLIA/CAP-accredited laboratory (Caris Life Sciences, Phoenix, AZ, USA), which interrogates coding regions of cancer-related genes. The assay covers single-nucleotide variants, insertions/deletions, and selected copy number alterations. Sequencing was performed with an average coverage depth of >500×, ensuring high sensitivity for mutation detection. The panel includes key oncogenes and tumor suppressor genes relevant to colorectal cancer. Orthogonal validation using Sanger sequencing was not performed due to the retrospective design and limited availability of residual tumor material. However, all NGS analyses were conducted in a CLIA/CAP-accredited laboratory with validated pipelines, ensuring high analytical accuracy. Data from 17 patients were further analyzed, and one patient did not undergo NGS due to the anticipated out-of-pocket cost, as documented in the clinical record.

### 2.3. Immunohistochemistry Staining of SMAD4

Sections (4 μm) were cut from paraffin blocks and deparaffinized using standard methods. Slides were treated with sodium citrate and steamed for 30 min at 80 °C. After cooling for 5 min, the slides were labeled with a monoclonal antibody specific to Smad4 (Clone D3R4N, Cell Signaling Technology, Inc. Danvers, MA, USA) at a dilution of 1:400. The antibody was detected using a biotinylated secondary antibody and 3,3′-diaminobenzidine as the chromogen. Then, the slides were counterstained with hematoxylin.

All SMAD4 immunohistochemical evaluations were performed independently by pathologists who were blinded to clinical, molecular, and outcome data to minimize potential bias. SMAD4 expression was evaluated semi-quantitatively using both staining intensity and distribution. Expression patterns were categorized as follows: (1) retained/standard expression (comparable to adjacent normal mucosa), (2) strong expression (>95% tumor cells with strong intensity), (3) weak expression (reduced intensity compared to normal mucosa), and (4) loss of expression. Loss was defined as the complete absence of staining in ≥5% of tumor cells. Clonal loss was defined as the complete loss of SMAD4 staining in cohesive groups of tumor glands or clusters, while surrounding tumor cells retained expression, indicating intratumoral heterogeneity [12].

### 2.4. SMAD4 Protein Alterations and Clinicopathological Characteristics

To investigate the association between SMAD4 protein alteration and clinicopathological characteristics, 10 variables were examined across all 18 patients based on pathological reports and clinical records (Table 2).

### 2.5. Statistics Analysis

Associations between SMAD4 alterations and clinicopathological variables were assessed using Fisher’s exact test. Survival curves were generated using the Kaplan–Meier method and compared with the log-rank test. *p*-values < 0.05 were considered statistically significant.

## 3. Results

### 3.1. Clinical Features of the EOCRC < 40 Years Old Cases

The clinicopathologic findings for the 18 cases are detailed in Table 1. A comprehensive review of the clinical features revealed a balanced distribution between males (8/18) and females (10/18). The age range of the patients spanned from 17 to 38 years, with a mean age of 32.7 years and a median age of 34 years. The primary tumor sites exhibited a predominant presence in the rectum (10/18, 56%), followed by tumors located in the sigmoid colon (3/18, 17%) and the descending colon (3/18, 17%). Singular cases were found in the transverse and ascending colon, respectively.

The diagnosis was typically made at an advanced stage (T3 or T4) in 12 out of 18 cases (67%), with most tumors being moderately differentiated (G2) in 14 out of 18 cases (78%). Case #1, involving the youngest patient in this cohort at 17 years old, exhibited high-grade features with signet-ring cells (G3). Lymph vascular invasion was observed in 50% of cases (9/18), with 89% of these cases showing small vessel invasion (8/9). Lymph node metastasis was detected in 39% of cases (7/18). Perineural invasion was identified in 50% of cases (9/18), underscoring the invasive nature of the tumors. Most cases were identified as microsatellite stable (MSS) and proficient in mismatch repair (MMR) protein, accounting for 16 out of 18 cases (89%). Small vessel invasion is associated with lymph node metastasis and has been shown to be an independent indicator of adverse outcomes in some studies [15,16,17]. Microsatellite instability-high (MSI-H) was detected in two cases, both of which involved germline mutations in MMR genes. No sporadic MMR-deficient cases were detected in this cohort.

### 3.2. Genomic Alterations Detected in the EOCRC < 40 Years Old Cases

The most common mutant genes in this study include KRAS (35%, 6/17), APC (24%, 4/17), TP53 (17.6%, 3/17), and SMAD4 (17.6%, 3/17). Genomic alterations of SMAD4 were focused on in this study. These SMAD4 alterations consisted of one missense mutation and two nonsense mutations. Notably, one of the identified nonsense mutations, c.1162C>T (p.Q388*, case #7), and the missense mutation 1082G>A (p.R361H, case #10), have been previously characterized as pathogenic mutations linked to JPS and CRC [9,18,19,20,21], and especially, the missense mutation p.R361H is a relative hotspot mutation of SMAD4 in CRC [22,23].

A novel nonsense mutation, p.E33* (case #4), was identified in this research, resulting in premature termination of transcription at the 33rd amino acid within the N-terminal domain of the SMAD4 protein. Normally, the structural integrity of the SMAD4 protein is governed by a highly conserved arrangement of two functional domains: the N-terminal MH1 domain responsible for DNA binding, and the C-terminal MH2 domain crucial for transcriptional activation, as elucidated by Qin B. et al. [24]. The truncation caused by this newly identified mutation likely renders the protein completely inactive, as it leads to the loss of essential MH1 and MH2 functional domains, compromising its biological function in the TGF-β signaling pathway.

It is noteworthy that all three SMAD4 genetic alterations we detected occurred exclusively in the subset of EOCRC cases harboring mutated KRAS genes, indicating a significant association (*p* = 0.0245). In contrast, no SMAD4 alterations were observed in the KRAS wild-type EOCRC cases (Supplement Table S1).

### 3.3. Protein Expression Alterations of SMAD4 in Early-Onset Patients Are More Significant than Genomic Mutations

**Figure 1:**
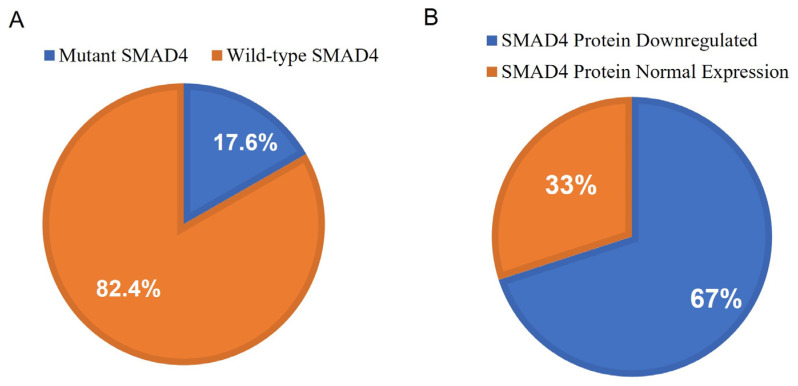
SMAD 4 protein expression downregulation is more significant than genomic mutation: (**A**) SMAD4 gene mutation and (**B**) SMAD4 protein expression status.

Immunohistochemistry staining of SMAD4 revealed a higher prevalence of downregulated protein compared to its genomic mutation rate, affecting 67% of cases (12 out of 18) (Figure 1). Among the SMAD4 wild-type cases, 60% showed downregulation at the protein level (9 out of 15).

**Figure 2:**
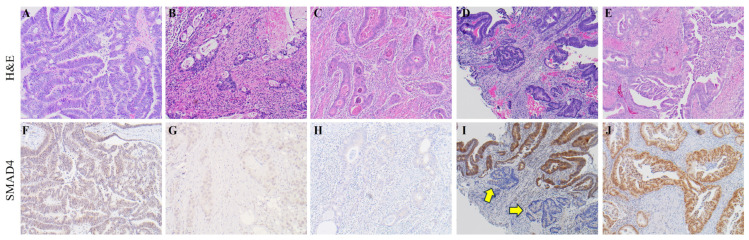
Morphology and SMAD 4 express patterns of EOCRC: (**A**–**E**) Representative images of hematoxylin and eosin staining (**E**,**H**); (**F**–**J**) immunohistochemistry of SMAD4; (**A**,**F**) standard SMAD4 expression, (**B**,**G**) weak expression, (**C**,**H**) complete loss, (**D**,**I**) clonal loss, and (**E**,**J**) strong nuclear expression. 20× Magnification. Yellow arrows indicate the tumor with SMAD4 clonal loss.

Five different SMAD4 expression patterns were identified in the 18 young CRC patients, including standard expression, weak expression, complete loss, heterogeneous loss (clonal loss), and strong nuclear expression by IHC staining (Figure 2).

Six patients showed standard expression (6/18), in which SMAD4 is typically located in both the cytoplasm and the nucleus, similar to normal mucosa; nine patients showed loss of SMAD4 expression, including complete (5/18) or clonal loss (4/18), whereas one patient showed strong, and two patients showed weak expression, respectively (Figure 2). The two patients with SMAD4 nonsense mutation (case #4 and case #7) showed SMAD4 protein loss, and the missense mutation case (case #10) showed strong nuclear staining (Table 1). Interestingly, 60% of patients with wild-type SMAD4 (9/15) showed loss or weakness of SMAD4 protein expression. These results suggest that post-translational mechanisms or epigenetic modifications might play a crucial role in SMAD4 protein dysregulation in EOCRC patients. In case #10, a p.R361H mutation was detected, which was reported as a high-prevalence mutation in CRC, and the mutant protein disrupted Smad2/3-Smad4 heteromeric complex formation and abolished canonical TGF-β signaling. In that, they were similar to SMAD4 loss [20], although IHC showed a strong positive.

### 3.4. Loss of SMAD 4 Protein Is Related to Lymph Node Metastasis

**Figure 3:**
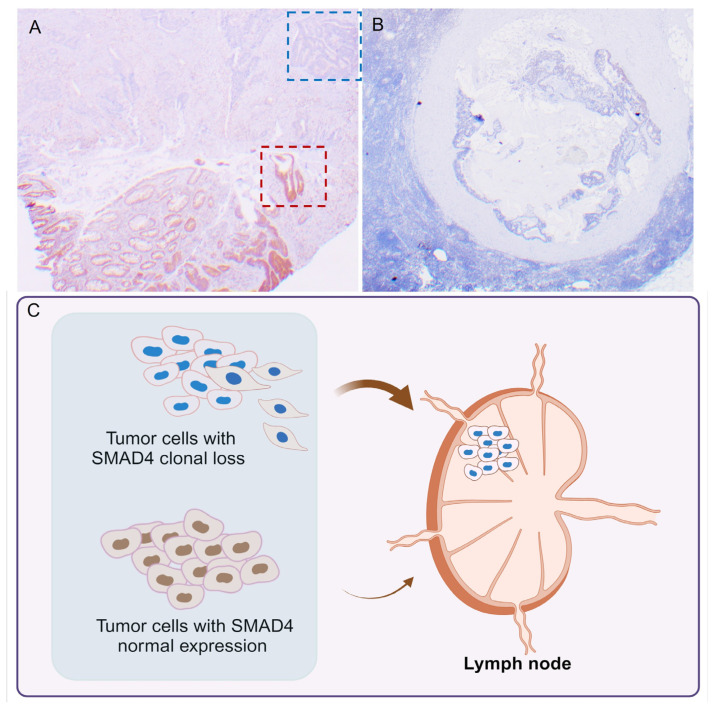
Loss of SMAD 4 expression is related to lymph node metastasis: (**A**) tumor section with SMAD4 protein intact (red box) and clonal loss of tumor cells (blue box); (**B**) lymph node with tumor metastasis from the same patient, showing negative for SMAD4. (**C**) Cartoon model illustrating that a subclone of tumor cells with SMAD4 loss preferentially metastasizes to the lymph node, whereas tumor cells retaining SMAD4 expression remain predominant in the primary tumor.

In this study, lymph node metastasis was more common in the group with downregulated SMAD4 protein (7/12) than in the standard SMAD4 group (0/6) (*p* = 0.0377). All cases with lymph node metastasis exhibited downregulated SMAD4 protein expression (Table 1 and Table 2). Interestingly, among the four cases with SMAD4 protein clonal loss, 50% had lymph node metastasis (2/4), and in those metastatic lymph nodes, only tumor cells negative for SMAD4 were detected (Figure 3A,B). This suggests that in patients with SMAD4 clonal loss, tumor cells lacking SMAD4 were more prone to metastasize to lymph nodes compared to cells with normal SMAD4 expression (Figure 3C).

### 3.5. Follow-Up

**Figure 4:**
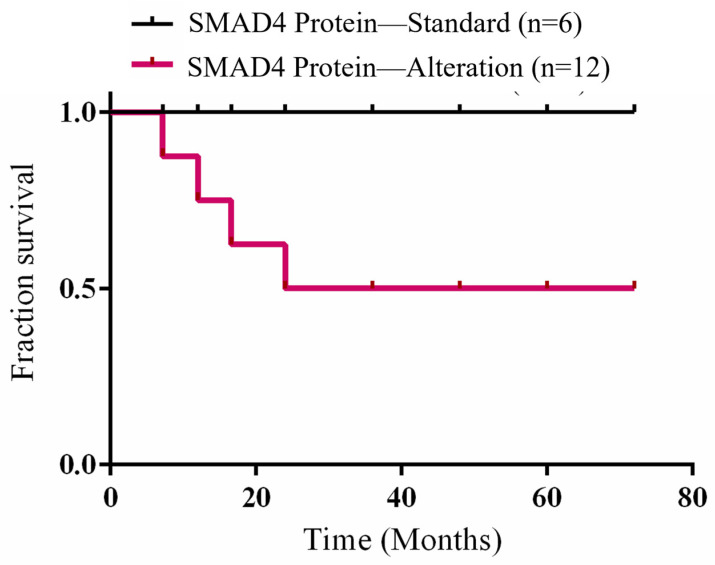
Survival curve of SMAD4 standard expression and downregulated EOCRC patients.

Further clinical follow-up data were obtained for 18 patients (median: 24 months; range 6.2–72.7 months). Excluding the two cases with pre-resection metastasis, 31% (5/16) developed post-resection metastases at intervals ranging from 5.7 to 32.6 months (median, 9.6 months), commonly to the lung (4/5, 80%), liver (2/5, 40%), and lymph nodes (2/5, 40%). Patient #7 experienced recurrence at 2.4 months, with an NGS test revealing new mutations in APC, B2M, TCF7L2, TERT, and TP53 in addition to the initially detected KRAS mutation. Currently, eight patients are alive with no evidence of disease, while four patients succumbed to the disease within 7.2 to 24 months (mean: 16.8 months) of diagnosis, all showing alterations in SMAD4 protein expression (*p* = 0.045, Figure 4). This result suggested that patients with SMAD4 protein alterations may be associated with poor survival, especially when in the proximal colon (ascending and transverse), resulting in a 100% (2/2) mortality rate within 18 months (16.6 months and 7.2 months). While a SMAD4 protein alteration was associated with poorer survival, these findings are preliminary rather than definitive. Larger studies with a longer follow-up are required to validate the prognostic significance of SMAD4 alterations in EOCRC.

## 4. Discussion

This study provides insights into the clinical and molecular characteristics of early-onset colorectal cancer (EOCRC) in a cohort of patients under 40 years old. The predominant tumor localization is in the rectum (56%). The advanced stage at diagnosis (67% at T3 or T4) emphasizes the aggressive nature of EOCRC compared to traditional late-onset colorectal cancer. These findings are consistent with existing literature that suggests EOCRC often presents with advanced disease [18,25].

In previous studies, it was found that in patients with EOCRC, MSI-H tumors were more commonly attributed to germline mutations in MMR genes [26,27,28] rather than to epigenetic inactivation of the MLH1 MMR gene, which is more common in the sporadic MSI-H tumors observed in patients with later-onset colorectal cancers [25,29]. These findings align with the results of our study, too.

The SMAD4 tumor suppressor gene product is important in intestinal carcinogenesis. Germline mutations in SMAD4 cause juvenile polyposis syndrome (JPS) with a dominantly autosomal inherited predisposition to multiple gastrointestinal polyps and cancer [9]. SMAD4 mutations have recently been reported in 5–20% of sporadic colorectal carcinomas (CRC), where they were associated with distant metastases and/or poor prognosis in some studies but not in others [22,30,31,32,33,34]. The SMAD4 gene is located on chromosome 18q21.1 and spans 55,000 base pairs across 11 exons, encoding a 551-amino acid protein. It contains functional domains, including the MH1 domain responsible for DNA binding, a domain that connects the two MH domains, and the MH2 domain crucial for homodimerization, heterodimerization, transcriptional activation, and the nuclear localization of SMAD4 [24]. SMAD4 genomic alterations were detected in 17.6% of cases in our study, including previously characterized pathogenic mutations linked to juvenile polyposis syndrome, and we also identified a novel nonsense mutation, p.E33*, to the spectrum of SMAD4 mutations, which likely results in complete functional loss of the protein due to the truncation of essential domains. The SMAD4 genomic mutation rate is consistent with previous studies, which show that there were no significant differences in genomic tumor profiles between patients with early-onset and those with later-onset colorectal cancer [35].

Furthermore, the strong association between SMAD4 mutations and KRAS mutations suggests a potential synergistic role in EOCRC pathogenesis, which is consistent with previous findings that highlight the interplay between these genes in colorectal cancer [9,30]. In normal cells, SMAD4 is typically located in both the cytoplasm and the nucleus, with its localization changing upon activation by TGF-β signaling. Loss of SMAD4 expression has been observed in 37% of primary CRC cases in individuals over 40 years old [15]. Another study reported a 19% rate of SMAD4 protein alterations in late-onset CRC patients, which is generally interpreted as a sign of a poorer prognosis due to the loss of tumor-suppressive activities associated with TGF-β signal transduction [13]. The diverse expression patterns of SMAD4 in EOCRC, including complete loss, clonal loss, weak expression, and strong nuclear expression, highlight the complex regulatory mechanisms governing this protein. In our EOCRC under 40 years old cohort, the higher frequency of SMAD4 protein downregulation compared to genomic mutations (67% vs. 17.6%) suggests additional regulatory mechanisms. Potential explanations include epigenetic silencing, such as promoter methylation, microRNA-mediated suppression, and post-translational modifications leading to altered protein stability or degradation. Furthermore, disruptions in TGF-β signaling components may affect SMAD4 nuclear localization and function without altering protein expression levels detectable by sequencing. These mechanisms may together contribute to SMAD4 dysregulation in EOCRC. This finding indicates that SMAD4 immunohistochemistry (IHC) may be more critical to perform than genomic mutation analysis in EOCRC patients In addition, SMAD4 p.R361H was reported as a functional loss and a hotspot mutation in CRC that shows diffuse strong nuclear positive in our immunohistochemistry staining, like the IHC pattern of some of the P53 mutation; therefore, we looked at it as “SMAD4 loss” [20].

Recent large-scale genomic and multi-omics studies, including TCGA-based and multi-institutional analyses, have demonstrated that EOCRC shares many molecular features with later-onset CRC while also exhibiting distinct clinicopathological characteristics [6,7,8,35]. Consistent with these reports, our study found no marked difference in the overall mutation frequency of key driver genes such as KRAS, APC, and TP53. However, our findings highlight a disproportionately higher rate of SMAD4 protein downregulation relative to genomic alterations, suggesting that post-genomic regulatory mechanisms may play a more prominent role in EOCRC. This observation complements prior multi-omics studies by emphasizing the importance of protein-level evaluation in understanding tumor biology.

The correlation between SMAD4 protein alterations and lymph node metastasis (58% in cases with downregulated SMAD4 vs. 0% in cases with standard expression) supports the hypothesis that SMAD4 plays a critical role in tumor invasiveness and metastasis. This is further supported by the observation that clonal loss of SMAD4 protein is associated with a higher propensity for lymph node metastasis, indicating that SMAD4-deficient tumor cells have an enhanced metastatic potential as reported in previous studies [13,15].

Follow-up data reveal that post-resection metastases occurred in 31% of patients, predominantly affecting the lungs, liver, and lymph nodes, with a median time to metastasis of 9.6 months. This high rate of metastasis underscores the aggressive nature of EOCRC. The particularly poor prognosis for EOCRC cases in the proximal colon aligns with previous studies indicating a worse outcome for right-sided colorectal cancers [19].

Our study has several limitations. Firstly, the relatively small sample size may compromise the generalizability of the findings to a larger population. This study should be interpreted as an exploratory analysis given the relatively small cohort size, which reflects the rarity of colorectal cancer in patients under 40 years of age. Although all eligible cases within the study period were included to minimize selection bias, the limited sample size restricts the statistical power and generalizability of the findings. Larger, multi-institutional studies are needed to validate these observations. Secondly, as the study was conducted at a single center, there is a risk that the lack of diversity in the patient population could introduce bias into the results. Thirdly, this study did not compare early-onset colorectal cancer cases to later-onset cases, which hinders the ability to make direct comparisons and understand the unique characteristics of early-onset colorectal cancer, although comparisons were made with previously published later-onset cases. Fourthly, the follow-up period was relatively short, potentially overlooking long-term outcomes and recurrence patterns in early-onset colorectal cancer patients. Additionally, given the limited sample size and number of outcome events, multivariate analysis was not performed due to the risk of model overfitting and unreliable estimates. Future studies with larger cohorts are needed to determine whether SMAD4 protein alterations are independently associated with metastatic behavior.

## 5. Conclusions

This study highlights the unique clinical and molecular landscape of EOCRC < 40 years old, emphasizing the aggressive nature of the disease and the significant role of SMAD4 protein expression alterations. The findings suggest that SMAD4 protein downregulation, more prevalent than genomic mutations, is a critical factor in tumor progression, metastasis, and poor prognosis. The association between SMAD4 and KRAS mutations provides a potential pathway for targeted therapies. The poor prognosis associated with SMAD4 protein alterations and proximal colon tumors underscores the need for early detection and aggressive treatment strategies in EOCRC < 40 years.

Future research should focus on elucidating the mechanisms underlying SMAD4 protein downregulation and its interaction with other oncogenic pathways. Additionally, exploring targeted therapies that address the SMAD4 and KRAS pathways may offer new hope for improving outcomes in EOCRC patients.

## Figures and Tables

**Table 1:** Clinicopathologic and molecular features of 18 patients with EOCRC < 40 years old.

Case #	Age	Gender	Ethnic	MMR Status	MSI	T	N	M	Grade	Location	LV Inv	PN Inv	Current Status	SMAD4 Gene	SMAD4 Protein	Other Pathogenic Mutations
1	17	M	AA	Intact	MSS	T4	N2a	M0	G3	Ascending	P (S)	Present	DOD (16.6 mo)	WT	Complete loss	TP53, FGFR2
2	24	F	W	Intact	MSS	T4b	N1b	M1	G2	Descending	P (S)	Present	AWM	WT	Weak	KRAS
3	34	F	AA	Intact	MSS	T1	N1a	M0	G1	Sigmoid	P (S)	Present	AWOD	WT	Clonal loss	KRAS
4	37	F	W	Intact	MSS	T4b	N0	M0	G1	Rectum	NI	Present	AWM	MUT (p.E33*)	Clonal loss	KRAS, APC, B2M, TCF7L2, TERT, TP53
5	34	F	AA	Intact	MSS	T2	N0	M0	G2	Descending	P (S)	NI	AWOD	WT	Clonal loss	NBN
6	36	M	W	Intact	MSS	T3	N0	M0	G2	Rectum	P (S)	Present	AWOD	WT	Standard	ND
7	37	M	W	Intact	MSS	T4b	N0	M0	G2	Descending	P (S)	NI	AWM	MUT (p.Q388*)	Complete loss	APC, KRAS
8	30	M	W	Intact	MSS	T2	N1b	M0	G2	Rectum	P (S)	Present	AWOD	WT	Clonal loss	ND
9	30	M	W	Intact	MSS	T2	N0	M0	G2	Rectum	NI	NI	AWOD	WT	Standard	APC, ERBB2, TP53
10	35	F	AA	Intact	MSS	T3	N0	M0	G2	Transverse	P (S and L)	Present	DOD (7.2 mo)	MUT (p.R361H)	Strong	BCOR, BRAF, KRAS, PI3K, RSPO3
11	31	F	W	Intact	MSS	T1	N0	M0	G2	Rectum	NI	NI	AWOD	WT	Standard	ND
12	29	M	W	Intact	MSS	T4b	N1	M1a	G2	Sigmoid	NI	NI	DOD (24 mo)	WT	Complete loss	APC, FBXW7, KRAS, PIK3CA, RET
13	32	F	W	Intact	MSS	T2	N1a	M0	G2	Rectum	NI	NI	AWM	WT	Complete loss	ND
14	38	F	AA	Intact	MSS	T3	N0	M0	G2	Rectum	NI	Present	AWOD	WT	Standard	ND
15	37	M	W	Intact	MSS	T3	N0	M0	G1	Rectum	P (L)	NI	AWOD	WT	Standard	BRCA2
16	34	M	Other	intact	MSS	T3	N0	M0	G2	Rectum	NI	NI	DOD (11.9 mo)	NA	Complete loss	NA
17	36	F	AA	MHL1, PMS2 Loss	MSI-H	T3	N1a	M0	G2	Rectum	NI	Present	AWOD	WT	weak	PMS2
18	38	F	W	MSH2 Loss	MSI-H	T3	N0	M0	G2	Sigmoid	NI	NI	AWOD	WT	Standard	MSH2

* Denotes a premature stop codon.

**Table 2:** SMAD 4 gene and protein status and other clinicopathologic features.

		SMAD4 Expression		
		Standard n (%)	Altered n (%)	*p* Value
SMAD4 Gene				
	WT	6 (35.4)	8 (47)	*0.272*
	Mutant	0 (0)	3 (17.6)	
Gender				
	M	2 (11.1)	6 (33.3)	*0.638*
	F	4 (22.2)	6 (33.3)	
Location				
	Right	0 (0)	2 (11.1)	*0.529*
	Left	6 (33.3)	10 (55.6)	
T stage				
	T1, T2	2 (11.1)	4 (22.2)	*1.000*
	T3, T4	4 (22.2)	8 (44.4)	
N stage				
	N0	6 (33.3)	5 (27.8)	* *0.0377*
	N1, N2	0	7 (38.9)	
M stage				
	M0	6 (33.3)	10 (55.6)	*0.529*
	M1, M2	0	2 (11.1)	
Histology grade				
	G1, G2	6 (33.3)	11 (61.1)	*1.000*
	G3	0	1 (5.6)	
Lymphovascular invasion (S)				
	Absence	5 (27.8)	5 (27.8)	*0.152*
	Presence	1 (5.6)	7 (38.9)	
Lymphovascular invasion (L)				
	Absence	5 (27.8)	11 (61.1)	*1.000*
	Presence	1 (5.6)	1 (5.6)	
Perineural invasion				
	Absence	4 (22.2)	5 (27.8)	*0.619*
	Presence	2 (11.1)	7 (38.9)	
Microsatellite instability				
	Stable	5 (27.8)	11 (61.1)	*1.000*
	High	1 (5.6)	1 (5.6)	

* Indicates statistical significance (*p* < 0.05).

## Data Availability

The original contributions presented in this study are included in the article/Supplementary Materials. Further inquiries can be directed to the corresponding author.

## Supplementary Materials

The following supporting information can be downloaded at: https://www.mdpi.com/article/10.3390/diagnostics16121804/s1, Table S1: SMAD4 genetic mutation occurred exclusively in the subset of EOCRC cases harboring mutated KRAS genes.

## Abbreviations

The following abbreviations are used in this manuscript:

F	Female
M	Male
AA	African American
W	White
NA	Not available
NI	Not identified
ND	No pathologic mutation detected
S	Small vein
L	Large vein
P	Present
LV inv	Lymphovascular invasion
PN inv	Perineural invasion
WT	Wild type
MUT	Mutant
DOD	Died of disease
AWM	Alive with metastasis
AWOD	Alive without disease
