# Supplementary material for: SMAD4 Protein Alterations in Early-Onset Colorectal Cancer: Implications as a Potential Marker for Aggressive Disease and Prognosis—A Clinicopathological and Molecular Analysis of 18 Cases in Patients < 40 Years of Age
Source: Diagnostics (Basel). 2026 Jun 11;16(12):1804. doi: 10.3390/diagnostics16121804 (PMC13297952; doi:10.3390/diagnostics16121804)
Supplement: Supplementary file 1 [file diagnostics-16-01804-s001.zip › diagnostics-4289731-supplementary.pdf]

Supplementary Table S1. SMAD4 genetic mutation occurred exclusively in the subset of EOCRC cases harboring mutated KRAS genes

Results			
	SMAD4 mutant	SMAD4 wild-type	***Marginal Row Totals***
KRAS mutant	3	3	6
KRAS wild-type	0	12	12
Marginal Column Totals	3	15	18 (Grand Total)

Fisher exact test statistic P value is 0.0245. The result is significant at $p < 0.05$
